# Databases as policy instruments. About extending networks as evidence-based policy

**DOI:** 10.1186/1472-6963-7-200

**Published:** 2007-12-07

**Authors:** Antoinette de Bont, Herman Stoevelaar, Roland Bal

**Affiliations:** 1 ErasmusMC, Institute of Health Policy and Management, Postbox 1738, 3000 DR Rotterdam, The Netherlands

## Abstract

**Background:**

This article seeks to identify the role of databases in health policy. Access to information and communication technologies has changed traditional relationships between the state and professionals, creating new systems of surveillance and control. As a result, databases may have a profound effect on controlling clinical practice.

**Methods:**

We conducted three case studies to reconstruct the development and use of databases as policy instruments. Each database was intended to be employed to control the use of one particular pharmaceutical in the Netherlands (growth hormone, antiretroviral drugs for HIV and Taxol, respectively). We studied the archives of the Dutch Health Insurance Board, conducted in-depth interviews with key informants and organized two focus groups, all focused on the use of databases both in policy circles and in clinical practice.

**Results:**

Our results demonstrate that policy makers hardly used the databases, neither for cost control nor for quality assurance. Further analysis revealed that these databases facilitated self-regulation and quality assurance by (national) bodies of professionals, resulting in restrictive prescription behavior amongst physicians.

**Conclusion:**

The databases fulfill control functions that were formerly located within the policy realm. The databases facilitate collaboration between policy makers and physicians, since they enable quality assurance by professionals. Delegating regulatory authority downwards into a network of physicians who control the use of pharmaceuticals seems to be a good alternative for centralized control on the basis of monitoring data.

## Background

Several authors have stressed the need for improving evidence-based health policy [1-3]. One way of enabling truly evidence based policy is claimed to be the use of databases [4,5]. Where the results of clinical studies are lacking or are insufficient – for example in when there is uncertainty about the long-term effects of a specific technology – databases may be used to monitor the effectiveness and safety of using these technologies in clinical practice. In addition, monitoring can be used to produce data about the appropriateness of care and associated costs. In short, it is assumed that databases, particularly those linked to electronic medical records, would enable the long-term follow-up of patients, providing information about the diffusion of pharmaceuticals in clinical practice, their coverage and budget impact [6-8]. Several studies, however, indicate that data that are generated by monitoring systems are hardly used [9,10]. Lehenkari and Hyssalo, who studied a diabetes management system in Finland were confronted by a 'graveyard' of databases. From the 1980's on, 21 similar diabetes databases have been developed in Finland to monitor the incidence of retinopathy. Only four databases still exist (Lehenkari, J. and S. Hyysalo, Interventions in the networks of power and cooperative learning, submitted.). Likewise, Black and Paine, who conducted a survey of clinical databases in the UK, concluded that only a third of the databases had been used for research purposes [11]. In addition, Pollitt demonstrated in a recent literature review that the evidence of active and systematic use of performance data for policy purposes is limited [10]. However, considering the efficacy of databases only in terms of their use for policymaking may yield too limited a conceptual perspective. Pollitt suggests that the regular production of performance information may be as important as the consumption of these data [10]. Moreover, information technologies such as databases may have profound effects on the relation between clinicians, health-care managers, insurers and policy makers, as traditional patterns of governance seem to be challenged in the information age, creating new systems of surveillance and control [12-14]. According to Singh, information networks can be seen as governance networks that "allow for different forms of authority." Hence, databases may have a profound effect on controlling clinical practice, e.g. in facilitating collaboration between policy makers and physicians, even though they are hardly used in policy making as such.

From the 1990's the Dutch Health Insurance Board (CVZ) has been using clinical databases as instruments for evidence-based health policy making [6-8]. In 2004, the board of CVZ asked the Department of Health Policy and Management to evaluate the use of the databases to enforce policy measures, as the Board was dissatisfied with the databases they already had set up to control the use of a number of expensive drugs. According to them these databases did not contain the data they needed. The purpose of our study was to gain insight into the use of existing databases and to advise the board on whether and how they should use such databases to control the use of pharmaceuticals. To this end we studied the use of databases (both by policy makers and clinicians) for three expensive pharmaceuticals.

## Methods

We conducted a multiple case study. We selected three databases CVZ supported. These three were intended to be used to monitor the prescriptions of Taxol, growth hormone and antiretroviral drugs for HIV. The Taxol database was selected because CVZ saw it as a failure, while the other two databases were seen as a relative success. Although CVZ was not very satisfied with the content and the use of the growth hormone databases and anti-HIV database, they were at least seen as the best examples.

We started the case studies by reconstructing the development and use of the databases on the basis of the records of the CVZ. To study the use of the databases, we made an overview of relevant policy questions that were supposed to be answered with the data from the databases. In addition, we conducted 17 in-depth interviews with policy makers, researchers and physicians to understand why the databases were developed and how they were used or expected to be used. After the interviews, we organized two focus group sessions, which were conducted with a moderator and two observers. The focus groups were attended by physicians who developed and managed the databases, staff members of CVZ, representatives of health insurers and representatives of patient organizations. The topic guide for the focus groups was based upon the documents and the individual interviews. It contained the following issues: the use of databases, relevant policy questions and the possibilities to control the use of pharmaceuticals. Both the interviews and the focus groups were audio-taped and transcribed verbatim. The theoretical framework used to analyze the data was the sociotechnical perspective on IT [15,16]. This framework seeks to understand the interaction between technology and society. With this perspective we unraveled how the use of databases shaped the way the use of pharmaceuticals is controlled. We checked the results of our study with the CVZ and the owners of the databases.

### The context

In 2000, the Dutch government decided (after having taken many and increasingly detailed policy measures which did not have the expected cost reducing effects) to delegate the responsibility to control the use of pharmaceuticals to health insurers and health professionals [17]. The government realized that it could not control the use of and expenditure on pharmaceuticals exclusively through laws and regulation, and developed a series of new tools, such as protocols and databases, to promote rational prescribing.

These developments have led to new styles of regulation, of which conditional reimbursement is a good example. The goal of conditional reimbursement is to promote effective and efficient use of certain pharmaceuticals. For this purpose, reimbursement of a drug is made conditional on specific criteria or rules. For example, the application may be restricted to specific categories of patients or prescriptions may only be provided by authorized physicians. All drugs to which these conditions apply are included in the so-called Health Insurance Fund (Provision of Pharmaceuticals) Regulation Appendix 2. Criteria for inclusion are high costs, risk of inappropriate use, or the need of specific expertise to ensure appropriate patient selection [18]. The policy of conditional reimbursement was and still is considered a promising approach to the delivery of effective and efficient pharmaceutical care [19-21].

The reimbursement of Growth hormone, Taxol and antiretroviral drugs for HIV is conditional. They are only reimbursed if physicians follow a specific protocol. The reimbursement of the growth hormone and Taxol is also restricted to specific categories of patients. Taxol, for example, is only reimbursed for patients with ovarian or mama carcinoma with metastasis. For growth hormone, the first pharmaceutical that was labeled as an 'expensive pharmaceutical', a third condition is set. Prior authorization needs to be obtained from an expert committee before it can be reimbursed [19].

Between 1998 and 2004, the board of CVZ advised the Minster of Health seven times to provide reimbursement of a pharmaceutical under the condition that physicians collect data about its use and effects in daily practice. It was argued that the availability of data from real-life practice would provide the opportunity to control the use of pharmaceuticals. The Board wished to have up-to-date information about the use of these pharmaceuticals to check whether the guidelines for prescription were followed and to take new measures if necessary.

The databases we studied are presented as policy instruments, but they can best be compared with clinical databases, in that they were initiated by physicians who developed them for specific medical purposes. The Growth Hormone database and the antiretroviral drugs database were initiated by physicians to enable the long-term follow-up of patients, providing better insight into the effectiveness and adverse outcomes in clinical practice. Moreover, CVZ staff was not involved with the set-up of the databases. They became involved after the plans for the databases were already made. The Taxol database is an exception in this respect as the board was involved with this project from the start. However, the already existing infrastructure of the Dutch Clinical Cancer Database was used to develop the Taxol database. In short, the frameworks for these databases were established by medical associations.

## Results

### Providing information about the diffusion of pharmaceuticals in clinical practice

To gain insight into the use of the databases, we made an overview of the policy questions that were supposed to be answered with the information that would become available from the databases. On the basis of the study of the documents and the focus groups, we identified two sets of policy questions. The first set of questions concerned the effects of pharmaceuticals in daily practice, and the second focused on control of cost and quality.

In several policy documents the need is stressed for improving the evidence base for the reimbursement decision. It was suggested that the effects of pharmaceuticals might be different in clinical practice. As members of the Committee on Pharmaceutical Care of CVZ (who decide upon the reimbursement of pharmaceuticals) explained during interviews, trial data may not reflect clinical practice because they are collected in a controlled situation and over too short a period of time. Therefore databases were perceived as needed to monitor the effectiveness and safety of the use of pharmaceuticals in clinical practice.

The other recurring policy issue was controlling costs and quality. The CVZ staff was interested finding out whether physicians follow the protocols and/or whether new groups of patients get indicated for these pharmaceuticals. Working with the databases would allow CVZ staff to obtain more detailed information on medical practice. This is because in the case of the growth hormone database, and administrators visit the hospitals and retrieve the data directly from the medical records. The other databases also contain data from clinical records. In other words, the databases could provide CVZ staff with clinical data they otherwise would have no access to, as it would be inconceivable for CVZ staff to ask hospitals directly to open their medical archives in order to check the use of pharmaceuticals.

After reconstructing the main policy questions, we examined whether these policy questions were answered – whether data had been retrieved from the databases and analyzed to decide upon reimbursement or to check the actual use of the monitored pharmaceuticals.

It is difficult to reconstruct the decisions about the reimbursement, as the documents are not public. We have, however, some evidence that the data were not used. None of the key informants apparently knew whether the databases were used for decision making concerning coverage of either of these pharmaceuticals. Most of them believed that this had not been the case. According to the owner of the Taxol database, the board could not use the data for formal purposes as the data set was not yet complete at the time the decision about the reimbursement was made. Our document analyses confirmed that the data about the use of Taxol was published six months after the final decision about coverage was made [22,23].

In our interviews and in the focus group sessions, we asked CVZ staff members how they used the data to control the use of pharmaceuticals. They strongly doubted that is was possible to check the use of pharmaceuticals using these databases. The following quotes are typical examples of how the staff discussed the use of databases to control prescription behavior:

Respondent 1 "With a database we will know if things go wrong and we need to intervene. In order to do so we need to have extra instruments."

Respondent 2 "Yeah, the question is whether a database is of any use. What kind of evidence do we get from the data? Is it strong enough to make decisions about new interventions?"

Respondent 3 "I think monitoring is extravagantly promoted. It is nothing. It only means collecting data. That is all. It has become a bureaucratic duty that will take us a lot of time and cost a lot of money. And it is nothing."

Both types of policy questions, we can conclude, were not answered, at least not by the data collected in the database. Some data have been presented to policy makers. These reports were not made because the CVZ staff asked for them, but because an annual report was required. As one of our respondents said reporting data became "a bureaucratic duty."

### Databases and the governance of clinical practice

While the databases were not used to answer policy questions, they did nevertheless have a profound effect on controlling clinical practice as they facilitated collaboration between policy makers and physicians.

First, collecting data became an 'obligatory passage point' for treating patients with a certain drug [24]. As clinical databases became an instrument for evidence-based policy, CVZ started to support the development of these databases. They paid for the collection of the data. To enforce complete data sets, the registration of patients was made a condition for reimbursement. Before 1998 only academic centers in the western part of the Netherlands delivered data for the growth hormone database; after 1998 all physicians who wanted to treat patients with growth hormone had to participate in the database project. The HIV database, which was designed as a single centre database, was also extended into a national database on the basis of the CVZ policies. For Taxol treatments pharmacists needed a subsidy; otherwise the hospital had to pay for the drug itself. To receive the subsidy, they needed to deliver the clinical data to the centralized database.

Second, all drug-specific databases used explicit protocols for data collection, so that patients are treated and data are documented in a standardized manner. The guideline for using growth hormone is illustrative of this phenomenon [25]. In contrast to most professional guidelines in the Netherlands, this guideline does not start with a general description of growth hormone deficiency. Instead, it starts with a set of diagnostic criteria. The protocol provides a detailed description of how a physician should diagnose growth hormone deficiency and how this diagnosis should be checked before the treatment can start. The guidelines for Taxol and anti-HIV drugs function in a similar way, as do guidelines for using and monitoring specific pharmaceuticals. Again just like collecting particular data, adherence to these guidelines is a prerequisite for reimbursement.

As a result of the development of the databases these types of pharmaceuticals are only prescribed by specialized physicians. Although all general practitioners can basically prescribe anti-HIV drugs and all pediatricians are allowed to administer growth hormone, few actually do. Only a few general practitioners treat patients with AIDS because most patients are treated in specialized centers. Children with growth hormone deficiency are also treated less often in general hospitals by general pediatricians. Increasing numbers children with this deficiency are treated in specialized hospitals by specialized pediatricians. According to the administrators of the two databases for growth hormone and anti HIV drugs, the use of complex guidelines and registration forms made it more difficult (and cumbersome) for general practitioners or pediatricians in general hospitals to fulfill all requirements and to get paid for the drugs.

Paradoxically, the databases in this way enacted a policy that CVZ had wanted to implement earlier, but which was faced with serious physician opposition. CVZ wanted to restrict the prescription of growth hormone, Taxol, and anti HIV to only (highly) specialized physicians. The physicians, however, had strong objections to such a condition. According to the professional medical associations, physicians should be free to prescribe all drugs. Moreover, it would be difficult to define criteria for what constitutes a specialized physician. However, with the introduction of databases, an informal process of specialization did occur.

In effect, the deployment of databases can be seen as part of the establishment of interdisciplinary networks around the controlled use of specific drugs. The anti HIV drug database is a case in point [26]. In an internal evaluation report the possibilities of the database were praised in this respect. "According to the Health Care Insurance Board, the (...) project went very well. Thanks to this project, a network of physicians, pharmacologists and virologists for the introduction of the anti-HIV drug therapy was established (...). The Board wishes this network to continue [26]." Within the network CVZ is referring to in this quote, different professionals participate in this network CVZ is referring to in this quote: physicians, pharmacologists and virologists. They do not share a professional background, and they do not work for the same organization either. They are part of the network called the 'Network for monitoring the effect on HIV-1 infection of anti-retro-viral treatment', because they prescribe, deliver and check certain anti-HIV drugs. Not the patients they treat, but the drugs they use for the treatment of these patients, and in effect the data they collect for the central database, are the major link between them.

Similar networks have been established around growth hormone. All Dutch pediatrician endocrinologists meet four times a year in the Advisory group Growth Hormone (AGH). On their request, the National Registration of Growth hormone (LRG) analyses the database data. The LRG for example compared all patients with a partial growth hormone deficiency and reported the clinical results of these treatments. Since 1998 such an analysis has been the basis for several revisions of the guideline. Draft revisions of the guideline are discussed with all pediatrician-endocrinologists in the Netherlands during their annual meetings. The purpose of these discussions is to reach consensus. If consensus is reached, all pediatrician-endocrinologists receive an update of, or a supplement to the guideline [19].

As the example of database use shows CVZ is not the exclusive or dominant site for controlling the use of a certain drug as the deployment of databases supports the establishment of interdisciplinary networks around a specific drug. In all three networks around the drugs we studied, specific guidelines were used to check if the pharmaceuticals were prescribed appropriately. Moreover, as a result of the use of complex guidelines and registration forms, these types of pharmaceuticals are prescribed only by specialized physicians. In the networks using these protocols, collecting data and monitoring the treatment can hardly be separated.

## Discussion

Policy makers hardly use databases. CVZ staff asked for an infrastructure to collect data about the use of pharmaceuticals in daily clinical practice to check the use of pharmaceuticals and to make final decisions about reimbursement, but they never used the data for these ends. We demonstrated that the policy questions for which these databases were established needed no answers databases could provide. Policy questions are formulated when a new database is started, but these questions are forgotten the moment the data can be analyzed when the budget period is over. Rather than setting up or subsidizing databases to answer specific policy questions, it seemed that the mere existence of databases was considered enough from the perspective of policy, i.e. the function of databases from a policy perspective at first sight is symbolic rather than substantial (see also [27]).

However, as we showed in this paper, it is too simple to conceive the 'use' of databases only as retrieving and analyzing data to answer policy questions. The (policy) questions needed no answers because by developing these databases the regulatory authority was, albeit implicitly, delegated downwards into the network. The deployment of databases supported the establishment of interdisciplinary networks around a specific drug. In all three networks around the drugs we studied, specific guidelines were used to check if the pharmaceuticals were only prescribed to certain well-defined patient groups. Moreover, as a result of the use of complex guidelines and registration forms, these types of pharmaceuticals are prescribed only by specialized physicians. Thus, the databases and the protocols that form part of them are fulfilling control functions that were formerly located within the policy realm. In this sense, the databases studied here form typical examples of how information networks become governance networks as these networks form mechanisms of co-governance and self-governance [13].

Delegating regulatory authority solved important problems for CVZ. As policy makers explained to us, it is difficult for them to reconsider the reimbursement of a pharmaceutical on the basis of clinical data. In comparison to clinical trial data, still seen as the most important evidence for policy decisions, the evidence would simply not be strong enough. Retrospectively, the delegation of regulatory authority was a better, albeit implicit, strategy than take top-down measures based upon the data out of the database. A database can be an effective policy instrument as CVZ, together with physicians, succeeded in incorporating more and more physicians in their network (compare Bang [12]). By establishing databases, CVZ was able to check the use of pharmaceuticals in that they achieve a degree of compliance from physicians.

Databases are considered to be a new type of instrument in the progress of 'evidence-based' health policy [1-3]. Usually, the notion of evidence-based policy is framed to indicate that policymakers should make more use of the results delivered to them from science [4,5]. The idea that science and policy should be considered as separate realms, the one concerned with the production of objective truth and the other with the production of normative policies, has proved not to be an adequate description of actual policy-making practices (see e.g. [28,29]). Our analysis of the use of databases in Dutch pharmaceutical policy adds to these insights by showing that their effect was not so much tied to the data that were delivered to the policy arena, but rather to the mechanism for data collection and the way (the organization of) the databases changed the relations between clinical and policy practices. Rather than evidence leading to policy, it is the extension of the networks through which data flow (or do not flow) that seemed to affect the clinical practice of drug prescriptions. Taking into account these network characteristics seems to be a promising avenue for the further analysis of evidence-based policy.

## Conclusion

The databases we studied facilitate collaboration between policy makers and physicians, as they enable quality assurance by national bodies of professionals. The databases are fulfilling control functions that were formerly located within the policy realm. Delegation of regulatory authority downwards into a network of physicians who control the use of pharmaceuticals, formed around and as an effect of the databases, seems to be more effective than centralized control of results on the basis of monitoring data.

## Competing interests

The author(s) declare that they have no competing interests.

## Authors' contributions

AB carried out the case studies and drafted the manuscript. HS participated in conducting the case studies and helped to draft the manuscript. RB helped to draft the manuscript. All authors read and approved the final manuscript.

## Pre-publication history

The pre-publication history for this paper can be accessed here:


